# Efficacy of Biofeedback and Electrostimulation-Assisted Pelvic Floor Muscle Training between Women with Mild and Moderate to Severe Stress Urinary Incontinence

**DOI:** 10.3390/jcm11216424

**Published:** 2022-10-29

**Authors:** Jiun-Chyi Hwang, Fang-Ju Sun, Tsung-Hsien Su, Hui-Hsuan Lau

**Affiliations:** 1 Department of Medicine, Mackay Medical College, New Taipei 252, Taiwan; 2 Devision of Urogynecology, Department of Obstetrics and Gynecology, Mackay Memorial Hospital, Taipei 104, Taiwan; 3 Department of Medical Research, Mackay Memorial Hospital, Taipei 251, Taiwan; 4 Mackay Medicine, Nursing and Management College, Taipei 251, Taiwan

**Keywords:** biofeedback, electrical stimulation, electromyography, stress urinary incontinence

## Abstract

Background: To evaluate the efficacy of biofeedback and electrical stimulation-assisted pelvic floor muscle training (PFMT) between women with mild and moderate to severe stress urinary incontinence (SUI). Methods: This retrospective cohort study was conducted at a single center from 2014 to 2021. We included 57 patients with urodynamically proven SUI who underwent a biofeedback and electrical stimulation-assisted PFMT. They were categorized into mild and moderate to severe SUI. One-hour pad test from 2 to 10 g was defined as mild SUI, and ≥11 g was defined as moderate to severe SUI. Results: Fifty-seven patients were reviewed during the study period. Incontinence-related symptoms of distress, including the UDI-6, ISI, and VAS, all significantly improved in the mild SUI group (*p* = 0.001, *p* = 0.001 and *p* = 0.010, respectively), while only UDI-6 and VAS statistically improved in the moderate to severe SUI group (*p* = 0.027 and *p* = 0.010, respectively). There was significant improvement in IIQ-7 in the mild SUI group during serial treatments, but only in Session 6 in the moderate to severe SUI group. After 18 sessions of treatment, the UDI-6, ISI, and IIQ-7 scores showed significantly greater improvements in the mild SUI group compared to the moderate to severe SUI group (*p* = 0.003, *p* = 0.025, and *p* = 0.002, respectively). Conclusions: Although biofeedback and electrical stimulation-assisted PFMT is an effective treatment option for SUI, it is more beneficial for patients with mild SUI and a 1-h pad weight ≤ 10 g urine leak.

## 1. Introduction

Stress urinary incontinence (SUI) is defined as involuntary urine leakage during physical exertion, coughing, or sneezing [1]. The prevalence of SUI was reported to be 46% among adult women in the United States from 2005 to 2018 [2]. SUI is not an uncommon disorder and has negative impacts on quality of life. The first-line treatment for SUI is lifestyle modification and pelvic floor muscle training (PFMT) [3]. The principle of PFMT is to rehabilitate the denervation and weakness of the pelvic floor muscle and rebuild the functional integrity of the pelvic floor. The ultimate goal is to decrease urinary incontinence by increasing muscular tone; however, approximately half of patients fail this treatment and may opt for surgical interventions, such as a mid-urethral sling [3]. Failure to respond to treatment may be because more than 30% of women cannot contract their pelvic floor muscles correctly when performing PMFT [4].

The National Institute for Health and Care Excellence recommends biofeedback and/or electrostimulation as a first-line alternative to PFMT in patients with SUI who are unable to actively contract their pelvic floor muscles, or as a second-line treatment if PFMT alone is not sufficiently effective [5]. In biofeedback therapy, electronic instruments are used to relay auditory or visual information to help the patients be aware of the status of pelvic muscle activity. Electrostimulation is intended to stimulate the pudendal nerve and elicit contraction of the pelvic floor muscles, supporting the intrinsic part of the urethral sphincter-closing mechanism [5]. Although PFMT is used as the first-line management for SUI, the full potential of this treatment to cure or improve patients with mild and moderate to severe SUI is unclear. There are currently no agreed thresholds for the severity of SUI. If conservative treatment is ineffective in patients with more severe disease, it is necessary to inform them about the limitations and triage them directly to surgery to reduce healthcare costs and improve their experience.

In this study, we hypothesized that biofeedback and electrostimulation-assisted PFMT for women with moderate to severe SUI may be as effective as for those with mild SUI. To test this hypothesis, we investigated the effects of PFMT between women with mild and moderate to severe SUI by assessing subjective and objective outcomes. 

## 2. Materials and Methods

This retrospective cohort study was conducted at the Department of Obstetrics and Gynecology of a medical center in Taiwan from January 2014 to December 2021. All women underwent urodynamic examinations for a definitive diagnosis. We included patients who were diagnosed with urodynamic stress incontinence and underwent biofeedback and electrostimulation-assisted PFMT. The exclusion criteria were patients with an active vaginal or urinary tract infection, and those who were unable to contract their pelvic floor muscle due to cognitive deficits or neurological disorders. The women were categorized into two groups, those with mild SUI, and those with moderate to severe SUI. Mild SUI was defined as a 1-h pad test from 2 to 10 g. A 1-h pad test of more than 11 g was defined as moderate to severe SUI [6]. This study was approved by the Institutional Review Board of Mackay Memorial Hospital (14MMHIS031).

Biofeedback and electrostimulation-assisted PFMT was carried out by three physiotherapists. Every patient was given a leaflet with information on how to perform pelvic floor muscle exercises, and trainings were given twice a week, with six treatments as one course.

Before the first session began, pelvic floor anatomy was explained to the patients, and they were taught how to correctly contract pelvic floor muscles by the physiotherapists during every treatment session. At the beginning of each session, baseline vaginal contraction pressure was measured using a perineometer. Electromyography was recorded and analyzed using a Wireless Patient Module (Medical Measurement System, Enschede, The Netherlands). Two surface electrodes were attached at 3 and 9 o’clock directions from the anal sphincter to one channel. Another channel connected two surface electrodes, which were placed laterally to the umbilicus. Electromyography of pelvic floor muscle strength, including maximum voluntary contraction, maximal duration of sustained contraction, and synergic abdominal muscles, was measured during each contraction. The protocol of biofeedback was as follows: (1) fast contraction lasting for 1 s, rest for 2 s, repeated 15–20 times depending on the patient’s tolerance; and (2) rest for 1 min and then begin sustained contractions for 5 s, rest for 10 s, repeated 15–20 times. The duration of sustained contractions also depended on the patients’ tolerance.

Electrostimulation was performed using a FemiScan Pelvic Floor Therapy System with a Periform vaginal electrode probe (Mega Electronics, Kuopio, Finland) after biofeedback. The settings were frequency 35 Hz, pulse width 250 uS, stimulation time 5 s, and interval time 10 s. The output current was adjusted according to the patients’ maximal tolerable intensity (maximum 100 mA). The duration of electrostimulation was 20 min. After electrostimulation, the patients were taught to use an indicator stick connected to a vaginal probe, which provided visual feedback when the muscles were contracted correctly. Patients received instructions to perform pelvic floor muscle exercises with at least three sets of 10 to 15 repetitions a day at home before they left.

Evaluations included detailed medical history, pelvic examination, urine analysis, and 1-h pad test. Incontinence-related symptom distress and quality of life were assessed using the short forms of the Urinary Distress Inventory questionnaire (UDI-6), Incontinence Severity Index (ISI), visual analog scale (VAS), and Incontinence Impact Questionnaire (IIQ-7) at the first and final sessions of treatment, with higher scores indicating worse symptoms and poorer quality of life. The primary outcomes of interest were changes in UDI-6, ISI, VAS, and IIQ-7 scores between baseline and the last treatment session in the women with mild SUI compared to those with moderate to severe SUI. The secondary outcomes included electromyography changes in pelvic floor muscles, including maximal voluntary contraction, maximal duration of sustained contraction, and vaginal contraction pressure. 

Statistical analysis was performed using the independent *t*-test to evaluate the means of continuous variables. The chi-square test or Fisher’s exact test was used to evaluate between-group differences for categorical variables. For paired data at baseline compared with measurements after treatment, the paired *t*-test was used. A value of *p <* 0.05 was considered to be statistically significant. Statistical analysis was performed using the Statistical Package for the Social Sciences version 20 (IBM Corp., Armonk, NY, USA).

## 3. Results

A total of 57 women were included in this study from January 2014 to December 2021, including 36 with mild SUI and 21 with moderate to severe SUI. The demographic characteristics were similar between the two groups (Table 1). There were no significant differences in baseline incontinence-related symptoms of distress and baseline quality of life as evaluated by the UDI-6, ISI, VAS, and IIQ-7. Baseline electromyography measurements were recorded. The mild SUI group had a significantly higher vaginal contraction pressure compared with the moderate to severe SUI group (29.3 ± 14.8 vs. 20.9 ± 14.3 cmH_2_O, *p* = 0.039). No significant differences were noted in baseline maximal voluntary contraction and duration of sustained contraction. There was no significant difference in the total number of training sessions attended between the mild and moderate to severe SUI groups (13.7 ± 6.1 vs. 11.9 ± 6.4, *p* = 0.286).

Electromyographic measurements, incontinence-related symptoms of distress, and quality of life scores were compared between the first session of treatment (baseline) and the 6th, 12th, and ≥18th sessions in each group (Table 2). The mild SUI group had significant increases in maximal voluntary contraction (*p* = 0.006, 0.003 and 0.001, respectively) and duration of contraction (*p* = 0.002, 0.007, and <0.001, respectively) during each treatment sessions. In the moderate to severe SUI group, the increase in maximal voluntary contraction was not significant during each session, but the duration of contraction significantly increased at the 12th and ≥18th sessions (*p* = 0.056, 0.014, and 0.008, respectively).

There were significant decreases in the scores of UDI-6 (*p* < 0.001, <0.001, and 0.001, respectively), IIQ-7 (*p* = 0.002, 0.002, and 0.006, respectively), ISI (*p* < 0.001, 0.001 and 0.001, respectively), and VAS (*p* = 0.001, 0.001 and 0.010, respectively) after each session of treatment in the mild SUI group. In the moderate to severe SUI group, the scores of UDI-6 (*p* < 0.001, 0.026, and 0.027, respectively) and VAS (*p* < 0.001, 0.004, and 0.010, respectively) significantly decreased after each session of treatment. While the IIQ-7 score significantly decreased at the 6th session of treatment in the moderate to severe SUI group, there were no significant differences at the 12th and ≥18th sessions (*p* = 0.010, 0.197, and 0.070, respectively). There were no significant differences in ISI scores at the 6th, 12th, and ≥18th sessions in the moderate to severe SUI group (*p* = 0.061, 0.823, and 0.205, respectively).

After complete 18 sessions of biofeedback and electrostimulation-assisted PFMT, the women with mild SUI still had significantly higher vaginal contraction pressure compared to the women with moderate to severe SUI (39.8 ± 14.3 vs. 22.3 ± 8.8 cmH_2_O, *p* = 0.007) (Table 3). There were no significant differences in maximal voluntary contraction or duration of sustained contraction after treatment between the mild SUI and moderate to severe SUI groups. With regard to treatment success measured by UDI-6 (2.8 ± 2.6 vs. 7.7 ± 3.1, *p* = 0.003), IIQ-7 (2.4 ± 2.1 vs. 8.7 ± 5.1, *p* = 0.002), and ISI (1.8 ± 1.5 vs. 4.2 ± 2.7, *p* = 0.025), the mild SUI group had significantly better outcomes compared with the moderate to severe SUI group. No significant difference was noted in VAS score (2.7 ± 1.0 vs. 3.7 ± 1.0, *p* = 0.073).

## 4. Discussion

In the present study, we demonstrated that women with either mild or moderate to severe SUI both had significantly decreased incontinence-related symptoms based on UDI-6 and VAS after sessions of biofeedback and electrostimulation-assisted PFMT. However, with regard to incontinence-related quality of life and incontinence episodes, the women with moderate to severe SUI only had significant improvements after the first course of training, but then no further improvements in further treatment sessions. In contrast, the mild SUI group had continuous significant improvements; moreover, incontinence symptom distress, episodes of urinary leakage, and quality of life were significantly more improved after 18 sessions of treatment than in those with moderate to severe SUI.

During biofeedback, patients can learn to perform correct contraction patterns through visual and auditory feedback prompts, and reduce improper muscle usage patterns to increase pelvic floor muscle strength and endurance [7]. Electrostimulation has been shown to activate the efferent pudendal nerve stimulation through reflex activation and invigorate and strengthen muscle fibers with rapid contractions that are responsible for continence in situations of stress [8,9]. We observed significant gains in maximal voluntary contraction and duration of sustained contraction in the mild SUI group, but only a significant increase in the duration of sustained contraction in the moderate to severe SUI group. The mean fiber diameters in women with SUI are significantly smaller compared to those in asymptomatic women, with a tendency to have a lower number of type II muscle fibers [10]. Therefore, increased disease severity, possibly due to fibrosis and muscle fiber atrophy, may explain why maximal voluntary contraction did not significantly improve in the women with moderate to severe SUI after treatment in this study [11]. Strength training improves structural support, prolongs activation time, and enhances precontraction to raise intraurethral closure pressure, thereby reducing urine leakage [12]. Fast contraction may recruit type II muscle fibers to improve urethral response during straining [13]. Madill et al. demonstrated higher maximum voluntary pelvic floor muscle electromyography amplitudes in continent women compared to women with mild or moderate to severe SUI [14]. In addition, Shishido et al. found a higher vaginal pressure profile in continent women compared to those with SUI [15]. Consistent with these results, we also observed higher maximal voluntary contraction and vaginal contraction pressure in the mild SUI group both before and after treatment. This indicates that the severity of incontinence may be associated with pelvic floor muscle functional deficits. Reestablishing maximal voluntary contraction is difficult in patients other than in those with mild SUI, so the durability of long-term PFMT effects is uncertain in patients with moderate and severe incontinence. In this study, there was no significant increase in vaginal contraction pressure after treatment, possibly because it does not play a sufficiently specific or sensitive role in the continence mechanism. 

In 2021, a retrospective cohort study found that combining pelvic floor muscle exercises, functional electrostimulation, and timely biofeedback could decrease urinary leakage episodes and improve the quality of life in women with SUI [7], which is compatible with the present study. We found significant improvements in the quality of life in both the mild and moderate to severe SUI groups after treatment. However, while the improvement persisted in the mild SUI group after a series of treatment sessions, the improvement did not persist in the women with moderate to severe SUI. The ISI has been shown to be a valid and reliable method for determining the severity of incontinence [16]. There was no significant improvement in ISI in the moderate to severe SUI group in contrast to the mild SUI group. This may have had a negative impact on the quality of life [17] due to unfavorable treatment efficacy, which manifested as limited improvement on the IIQ-7. Some studies have investigated the factors which may be improved by PFMT in women with SUI. Cammu et al. found that physiotherapy was more likely to fail in women with two or more leakages per day [18]. They reported that 49% of the women considered their treatment to be successful, and that 51% experienced only some improvement, no change, or a worsening condition. They concluded the episodes of urine leak could predict therapy failure. Brooks et al. studied 77 women who completed a protocol of PFMT, of whom 38 (49%) were deemed to be cured. Among their patients, women with lower scores on the International Consultation on Incontinence Questionnaire Urinary Incontinence Short Form were more likely to have a dry pad test after PFMT than those with more severe symptoms [19]. They concluded that patients who had less severe incontinence symptoms were most likely to be cured with PFMT. This is similar to our results, as we found that PFMT resulted in a more promising treatment effect in the women with mild SUI.

The strength of this study is that we evaluated the outcomes with various valid questionnaires, and used the standard 1-h pad test, which is a widely used, noninvasive, semi-objective method for quantifying the severity of urine leak. To the best of our knowledge, this is the first study to compare the efficacy of biofeedback and electrostimulation-assisted PFMT between women with mild and moderate to severe SUI. Our results could provide valuable information for shared decision making. 

This study is limited by a small sample size and retrospective design. In addition, we only evaluated the effect at the final session of treatment, and longer trials of supervised PFMT are needed to evaluate the long-term efficacy.

## 5. Conclusions

Biofeedback and electrostimulation-assisted PFMT was beneficial for both the women with mild and moderate to severe SUI; however, the results were better in the women with mild SUI after treatment. Despite serial training sessions, the quality of life and symptoms of incontinence did not improve as much in the women with moderate to severe SUI compared to those with mild SUI.

## Figures and Tables

**Table 1:** Demographic characteristics of the women who underwent biofeedback and electrostimulation-assisted pelvic floor muscle training.

	Mild SUI (*n* = 36)	Moderate to Severe SUI (*n* = 21)	*p*
Age (y)	50.6 ± 11.3	50.3 ± 13.6	0.941
Parity (*n*)	2.2 ± 1.1	2.2 ± 0.9	0.821
Vaginal delivery (*n*)	30 (86%)	20 (95%)	0.393
Cesarean section (*n*)	6 (17%)	0 (0%)	0.074
Body mass index (kg/m^2^)	23.7 ± 2.8	23.4 ± 3.1	0.678
Hypertension (*n*)	6 (17%)	3 (14%)	>0.999
Diabetes (*n*)	3 (8%)	3 (14%)	0.659
Menopausal (*n*)	18 (50%)	9 (43%)	0.784
Previous incontinence surgery (*n*)	1 (3%)	1 (5%)	>0.999
Previous hysterectomy (*n*)	3 (8%)	1 (5%)	>0.999
1-h pad test (g)	2.5 ± 3.3	49.7 ± 35.5	<0.001 *
Baseline incontinence-related symptom distress and quality of life			
UDI-6	7.2 ± 3.4	8.6 ± 3.2	0.131
ISI	5.7 ± 4.5	5.9 ± 5.8	0.911
VAS	6.6 ± 5.0	6.2 ± 1.8	0.765
IIQ-7	8.0 ± 6.3	8.2 ± 4.4	0.880
Baseline electromyography measurements			
Maximal voluntary contraction (µV)	26.9 ± 14.8	22.8 ± 12.5	0.288
Duration of sustained contraction (s)	7.7 ± 4.8	6.4 ± 4.0	0.305
Vaginal contraction pressure (cmH_2_O)	29.3 ± 14.8	20.9 ± 14.3	0.039 *
Number of PFMT sessions (*n*)	13.7 ± 6.1	11.9 ± 6.4	0.286
Total duration of treatment (day)	72.7 ± 25.4	60.4 ± 38.1	0.227

Data are presented as mean ± standard deviation or as number (percent) of patients. SUI stress urinary incontinence, UDI-6 short form of the Urogenital Distress Inventory, ISI incontinence severity index, VAS visual analog scale, IIQ-7 short form of the Incontinence Impact Questionnaire, PFMT pelvic floor muscle training. * *p* < 0.05; significant difference between the women with mild and moderate to severe stress urinary incontinence.

**Table 2:** Comparisons of electromyography measurements and quality of life before and after treatment between groups.

	Number of Treatment Sessions	1st (Baseline)	6th	12th	18th or More	*p*		
						6th vs. Baseline	12th vs. Baseline	18th vs. Baseline
Mild SUI	Number of patients	36	34	26	19			
	Maximal voluntary contraction (µV)	26.9 ± 14.8	36.2 ± 28.7	39.7 ± 27.0	46.2 ± 26.2	0.006 *	0.003 *	0.001 *
	Duration of sustained contraction (s)	7.7 ± 4.8	11.4 ± 8.5	26.4 ± 36.7	48.5 ± 26.6	0.002 *	0.007 *	<0.001 *
	Vaginal contraction pressure (cmH_2_O)	28.9 ± 14.8	29.9 ± 14.3	34.8 ± 14.1	39.8 ± 14.3	0.595	0.061	0.069
	UDI-6	7.2 ± 3.4	5.8 ± 3.5	3.7 ± 2.7	2.8 ± 2.6	<0.001 *	<0.001 *	0.001 *
	ISI	5.7 ± 4.5	2.6 ± 1.7	2.1 ± 1.8	1.8 ± 1.5	<0.001 *	0.001 *	0.001 *
	VAS	6.6 ± 5.0	3.6 ± 1.9	2.8 ± 2.1	2.7 ± 1.	0.001 *	0.001 *	0.010 *
	IIQ-7	8.0 ± 6.3	5.6 ± 5.1	3.9 ± 4.	2.4 ± 2.1	0.002 *	0.002 *	0.006 *
Moderate to severe SUI	Number of patients	21	18	13	10			
	Maximal voluntary contraction (µV)	22.8 ± 12.5	23.6 ± 11.5	28.8 ± 17.0	37.0 ± 20.4	0.288	0.052	0.067
	Duration of sustained contraction (s)	6.4 ± 4.0	8.5 ± 4.0	17.0 ± 14.1	39.9 ± 24.3	0.057	0.014 *	0.008 *
	Vaginal contraction pressure (cmH_2_O)	20.9 ± 14.8	20.1 ± 16.4	24.5 ± 12.2	22.3 ± 8.8	0.802	0.063	0.487
	UDI-6	8.6 ± 3.2	5.3 ± 3.2	6.1 ± 4.1	7.7 ± 3.1	<0.001 *	0.026 *	0.027 *
	ISI	5.9 ± 5.8	2.6 ± 1.4	6.3 ± 12.2	4.2 ± 2.7	0.061	0.823	0.205
	VAS	6.2 ± 1.8	3.4 ± 1.2	2.9 ± 1.0	3.7 ± 1.0	<0.001 *	0.004 *	0.010 *
	IIQ-7	8.2 ± 4.5	5.5 ± 5.2	5.1 ± 5.6	8.7 ± 5.1	0.010 *	0.197	0.070

Data are presented as mean ± standard deviation. SUI stress urinary incontinence, UDI-6 short form of the Urogenital Distress Inventory, ISI incontinence severity index, VAS visual analog scale, IIQ-7 short form of the Incontinence Impact Questionnaire. * *p* < 0.05; significant difference between before and after pelvic floor muscle training.

**Table 3:** Outcomes after completing 18 sessions of biofeedback and electrostimulation-assisted PFMT.

	Mild SUI (*n* = 19)	Moderate to Severe SUI (*n* = 10)	*p*
Maximal voluntary contraction (µV)			
Final measurement	46.2 ± 26.2	37.0 ± 20.4	0.343
Change in measurement	20.1 ± 18.1	22.9 ± 23.2	0.759
Duration of sustained contraction (s)			
Final measurement	48.6 ± 26.2	39.9 ± 24.3	0.463
Change in measurement	42.7 ± 81.9	15.7 ± 11.5	0.400
Vaginal contraction pressure (cmH_2_O)			
Final measurement	39.8 ± 14.3	22.3 ± 8.8	0.007 *
Change in measurement	6.6 ± 13.4	3.3 ± 11.7	0.583
UDI-6			
Final measurement	2.8 ± 2.6	7.7 ± 3.1	0.003 *
Change in measurement	−3.7 ± 3.2	−2.8 ± 2.6	0.599
ISI			
Final measurement	1.8 ± 1.5	4.2 ± 2.7	0.025 *
Change in measurement	−4.1 ± 3.2	−6.6 ± 9.	0.605
VAS			
Final measurement	2.7 ± 1.0	3.7 ± 1.0	0.073
Change in measurement	−4.0 ± 5.3	−4.0 ± 2.3	0.976
IIQ-7			
Final measurement	2.4 ± 2.1	8.7 ± 5.1	0.002 *
Change in measurement	−4.1 ± 4.2	−3.4 ± 4.0	0.759

Data are presented as mean ± standard deviation. PFMT pelvic floor muscle training, SUI stress urinary incontinence, UDI-6 short form of the Urogenital Distress Inventory, ISI incontinence severity index, VAS visual analog scale, IIQ-7 short form of the Incontinence Impact Questionnaire. * *p* < 0.05; significant difference between the women with mild and moderate to severe SUI after pelvic floor muscle training.

## Data Availability

The data presented in this study are available on request from the corresponding author.
